# Anti-inflammatory and anti-fibrotic effects of intravenous adipose-derived stem cell transplantation in a mouse model of bleomycin-induced interstitial pneumonia

**DOI:** 10.1038/s41598-017-15022-3

**Published:** 2017-11-03

**Authors:** Takuya Kotani, Ryota Masutani, Takayasu Suzuka, Katsuhiro Oda, Shigeki Makino, Masaaki Ii

**Affiliations:** 1 Department of Internal Medicine (IV), Osaka Medical College, Osaka, Japan; 2 Division of Research Animal Laboratory and Translational Medicine, Research and Development Center, Osaka Medical College, Osaka, Japan; 3 Division of Central Laboratory, Osaka Medical College, Osaka, Japan

**Keywords:** Immunosuppression, Mesenchymal stem cells

## Abstract

Adipose-derived stem cells (AdSCs) have recently been considered a useful treatment tool for autoimmune disease because of their anti-inflammatory and immunosuppressive effects. We investigated the therapeutic effect of intravenous AdSC transplantation in a mouse model of bleomycin-induced lung injury. AdSCs accumulated in the pulmonary interstitium and inhibited both inflammation and fibrosis in the lung, markedly improving the survival rate of mice with bleomycin-induced lung injury in a cell number-dependent manner. AdSCs inhibited the production of pro-inflammatory cytokines such as TNF-α and IL-12 in activated macrophages, and AdSCs also induced the apoptosis of activated macrophages. AdSCs inhibited the differentiation and proliferation of Th2-type mCD4+ T cells but promoted the differentiation and proliferation of regulatory T cells, suggesting that the phenotypic conversion of T cells may be one of the mechanisms for the anti-inflammatory effect of AdSCs on pulmonary fibrosis. These findings suggest that intravenous AdSCs could be a promising treatment for patients with interstitial pneumonia.

## Introduction

Interstitial pneumonia (IP) is a life-threatening pathological condition that causes respiratory failure when it progresses. There are several types of IP: Fibrosis is the main pathology in usual IP (UIP), both interstitial inflammation and fibrosis develop in the lungs in non-specific IP (NSIP), and fibrosis development is induced by rapid pulmonary interstitial inflammation in diffuse alveolar damage (DAD)^1^. Lung inflammation is treated with steroids and immunosuppressants, and pulmonary fibrosis is treated with anti-fibrosis agents such as pirfenidone and nintedanib^1–4^. However, many cases are treatment-resistant and the outcome is poor in all disease types, which is problematic. Moreover, adverse effects such as infections resulting from immunosuppressive therapy are problematic. The development of new treatments is thus required for IP from the viewpoint of the poor effect and adverse effects of the currently available treatments.

Studies on mesenchymal stem cells (MSCs) derived from somatic tissues, such as bone marrow, skin, skeletal muscle, placenta, cord blood, and adipose tissue, have recently been actively performed in the field of regenerative medicine research^5–7^. Since MSCs have a potential to differentiate into other lineage cells, such as skeletal muscle cells, osteoblasts, chondrocytes, vascular endothelial/smooth muscle cells, and cardiomyocytes, the studies on treatment with cell transplantation are being performed aiming at clinical application^8,9^. In addition, MSCs have anti-apoptosis and anti-inflammatory actions, they modulate the immune response and adjust the microenvironment of the engraftment sites, and their efficacy against inflammatory and autoimmune diseases has been demonstrated^5,10–12^. Because expressions of human leucocyte antigen class I and II are low^5,6,10^ and immunological tolerance to intravenously administered MSCs has been shown^13^, they can also be used for allogeneic transplantation. It has been reported that systemic administration of bone marrow-derived MSCs resulted in cell accumulation in the pulmonary parenchyma and large airway in an animal model of pulmonary fibrosis prepared by airway administration of bleomycin (BLM) and reduced inflammatory cell infiltration and lung fibrosis^14–22^.

Among MSCs derived from different origins, adipose-derived stem cells (AdSCs) are advantageous because they can be easily isolated from fat tissue collected by liposuction, and the number of collectable cells per volume is higher than that with other types, e.g., in comparison with that in the same volume of bone marrow^23^. Research and development with AdSCs in immunosuppressive therapy have progressed specifically for graft versus host disease, Crohn’s disease, and Goodpasture syndrome, and favorable outcomes have been reported^24–26^. The inhibitory effects of AdSCs and AdSC-conditioned medium on pulmonary fibrosis induced by airway administration of BLM have also been reported^27–29^. However, precise histological examination of AdSC-recruited lung tissue, dose-dependent therapeutic effect of AdSCs, and the mechanism for the favorable effects of AdSCs has not been fully investigated in previous studies with a BLM-induced IP mouse model. IP induced by inhalation of BLM through the airway causes focal inflammatory/fibrotic lesions around bronchioles, which is not clinically compatible with IP in histopathology, and little is known about the precise mechanism for the effect of AdSCs on BLM-induced IP in mice. In contrast, the continuous subcutaneous infusion of BLM using an osmotic minipump forms inflammatory/fibrotic lesions mainly on pleural side, which is suitable for an IP model^30^.

In this study, we examined the therapeutic effect of intravenous AdSCs transplantation on BLM-induced IP mice in both inflammatory and fibrotic phases, and also investigated accumulation and retention of AdSCs in lung tissue, cell number-dependent therapeutic effects of AdSCs on lung inflammation, and the possible mechanism for the favorable effect of AdSCs on BLM-induced IP focusing on inflammatory cells which play an important role in IP.

## Methods

### Adipose Tissue Harvesting, AdSC Isolation, and AdSC Culture

The Institutional Animal Care and Use Committee of Osaka Medical College approved all of the following research protocols (approval ID: 27053), including the surgical procedures and animal care, and all methods were performed in accordance with the relevant guidelines and regulations. Female 13–14-week-old C57BL/6 J mice (SHIMIZU Laboratory Supplies, Kyoto, Japan) were sacrificed under anesthesia with pentobarbital (i.p., 200 mg/kg). Adipose tissue was harvested from inguinal areas and used in all experiments as subcutaneous white adipose tissue.

AdSCs were isolated from each adipose tissue sample as previously described with minor modifications^31^. Briefly, adipose tissue was washed in PBS and minced followed by digestion in 5 mL of type VIII collagenase (1 mg/mL in 1% BSA/HBSS) (Life Technologies Japan, Tokyo, Japan) for 40 min at 37 °C using a gentleMACS Dissociator (Miltenyi Biotec K.K., Tokyo, Japan) according to the manufacturer’s instructions. The digested tissue was filtered through a 40-μm cell strainer (BD Falcon, Tokyo, Japan) and centrifuged at 450 × *g* for 10 min. The supernatant containing adipocytes and debris was discarded. Pelleted cells were suspended with 5 mmol/L EDTA/PBS and layered over an equal volume of 1.083 g/mL Histopaque 1083 solution (Sigma-Aldrich Japan K.K., Tokyo, Japan). After centrifugation at 900 × *g* for 30 min, mononuclear cells (MNCs) were collected from the gradient interface. The MNCs were used as the freshly isolated adipose-derived stem/progenitor-rich cell (AdSC) population for the experiments. After reaching 70% confluence, cells were dissociated (0.25% trypsin EDTA; Invitrogen) and replated at 1000–3000 cells/cm^2^. Mouse AdSCs (mAdSCs) were cultured and used between the 3th and 4th passage in this study.

### Evaluation of mAdSC recruitment to lung

mAdSCs (2.5 × 10^4^) were labeled with 50 μg of rhodamine-conjugated poly (lactide-co-glycolide) (PLGA) for 6 h at 37 °C. After injection of BLM, the rhodamine-PLGA-labeled mAdSCs (Rho-mAdSC) (2.5 × 10^4^) were injected into BLM-treated mice via a tail vein on day 7. Mice were sacrificed 24 h after Rho-mAdSC injection, and the lungs were harvested for histological analysis. The Rho-mAdSCs were cultured *in vitro* and evaluated the rhodamine fluorescent signals after 1, 7, and 21 days by a flow cytometer (Cell Analyzer EC800, Sony, Tokyo, Japan). The fluorescent signals on day 7 (99.93%) and day 21 (99.98%) were similar to that on day 1 (Supplementary Fig. S1). The entire lung was sectioned with 40-µm and were examined under fluorescent microscope. The number of Rhodamine PLGA loaded –AdSCs (red fluorescent) was counted in all tissue sections, and the percent of the recruited AdSCs out of the intravenously infused AdSCs was calculated at 1, 7, and 21 days after mAdSC transplantation.

### Surgical Procedure and AdSC Transfusion Study

Female mice (C57BL6/J, 13–14 weeks old) were anesthetized with an i.p. injection of 400 mg/kg 2,2,2-tribromoethanol (Avertin, Sigma-Aldrich Japan K.K.). Mice were divided into 3 groups; BLM-alone, BLM-plus-mAdSC (2.5 × 10^4^), and BLM-plus-mAdSC (2.5 × 10^5^). Nine mice in each group were used for survival analysis and 5 for histological examination. The administered BLM was prepared by mixing sterile BLM sulfate powder (Nippon Kayaku, Tokyo, Japan) with sterile normal saline. A dose of 3 mg of BLM in a total volume of 100 μL of sterile saline was injected subcutaneously by osmotic minipump (Alzet 2010, DURECT, Cupertino, CA, USA) from day 0 to day 7. After the administration of BLM, mAdSCs were injected via a tail vein on day 7. Mice receiving the same volume of saline without mAdSCs were used for control. The mice were sacrificed at 7 and 21 days after mAdSC injection, and the lungs were harvested for histological analysis (Supplementary Fig. S2).

### Histological Analysis and Assessment of Fibrotic Area

The right middle lung of each mouse was fixed for 6 hours in 4% PFA/PBS followed by overnight incubation in 20% sucrose/PBS. The tissues were embedded in OCT compound (Sakura FineTek, Tokyo, Japan) and cut into 5-µm sections and stained with hematoxylin and eosin (H&E) or Masson’s trichrome stain for the analysis of morphological or fibrotic changes. For each slide, the 5 most severely injured, nonoverlapping fields (magnification × 200) of lung parenchyma were evaluated. Quantification of lung fibrosis on histological specimens was performed using a numerical scale (modified Ashcroft score)^32^. The severity of the fibrotic changes in each observed microscopic field of a given lung section was assessed and assigned a modified Ashcroft score from 0 to 8. Overall severity of each lung section was expressed as the mean of the scores of its observed microscopic fields. Histological examinations were assessed by 3 independent observers (MI, TS, KO). The blue area stained with Masson’s trichrome stain was determined as the fibrotic area. The fibrotic area/total area ratio was measured by a WinROOF Ver 6.1 computerized morphometry system (Mitani, Fukui, Japan) for each slide.

### Fluorescent Immunocytochemistry

The lung sections were washed with PBS, and the samples were blocked in antibody dilution buffer of 2% BSA/PBS for 15 min at room temperature (RT). After removal of the blocking solution, primary antibodies/markers were added to antibody dilution buffer at 37 °C for 2 h: isolectin B4 (ILB4) (1:100) (VECTOR Laboratories, Burlingame, CA) for endothelial cells; anti-CD3 (1:200) (eBioscience, San Diego, CA, USA) for T lymphocytes; anti-F4/80 antibody (1:200) (eBioscience) for macrophages; and Gr-1 (1:200) (eBioscience) for neutrocytes. After washing with PBS, cells were incubated for 30 min at RT with secondary antibodies prepared at 1:500 in antibody dilution buffer: Alexa 488 goat anti-rat IgG and Alexa 594 goat anti-rat IgG (Jackson ImmunoResearch Laboratories, Inc., West Grove, PA, USA). After the secondary antibodies were removed and the tissues were washed with PBS, nuclear counterstaining was performed by incubation with 4′,6-diamidino-2-phenylindole (DAPI) solution (1 µg/mL in PBS; Sigma-Aldrich Japan K.K.) for 10 min at RT. The sample slides were covered by cover slips with mounting medium (ImmunoBioScience, Mukilteo, WA) followed by sealing with nail varnish before evaluation under a fluorescence microscope (BZx-700, Keyence, Osaka, Japan). The positively stained cells in each sample were counted in 5 different high power fields (×200).

### Cell Function Assay

For murine CD4+ (mCD4+) T-cell function assay, thymus was harvested from anesthetized female mice (C57BL6/J, 13–14 weeks old), minced in 5 mmol/L EDTA-PBS, and filtered through a 40-μm cell strainer (BD Falcon, Tokyo, Japan) followed by centrifugation at 450 × *g* for 10 min. The supernatant containing debris was discarded. The pelleted cells were suspended with 5 mmol/L EDTA/PBS, and mCD4+ T cells were isolated from adherent cells by positive selection using a magnetic cell separation system (Miltenyi Biotec, CA, USA). The proliferation activity of mCD4+ T cells was examined using a Cell Counting Kit-8 (Dojindo Laboratories, Kumamoto, Japan) according to the manufacturer’s instructions. Briefly, mCD4+ T cells were seeded onto 96-well culture plates at a density of 1 × 10^4^ cells per well and cultured in RPMI containing 10% FBS or mAdSC condition medium (CM) containing 10% FBS stimulated with 0.25 μL/well of soluble anti-CD3/CD28 antibodies (Life Technologies AS, Oslo, Norway) and 0.2 μg/mL of recombinant murine IL-2 (PeproTech, Rocky Hill, NJ, USA) for 96 h at 37 °C. mAdSC CM was prepared by incubating mAdSCs with RPMI containing 1% FBS. Optical density was measured using a plate reader at a wavelength of 450 nm.

For the murine macrophages function assay, activated macrophages were isolated from female mice (C57BL6/J, 13–14 weeks old). Where indicated, peritonitis was induced by a single i.p. injection of 1 mL of 4% thioglycollate (Sigma, St. Louis, MO, USA) in PBS. At the indicated times after injection, the mice were sacrificed, and the abdominal cavities were flushed with 5 mL ice-cold PBS to harvest the cells. The isolated murine activated macrophages (5.0 × 10^4^) were directly incubated each with mAdSCs (5.0 × 10^4^, 1.0 × 10^5^, and 2.0 × 10^5^) in DMEM-F12 for 48 h. The attached cells were fixed with 4% PFA/PBS for 10 min at RT followed by washing with PBS. Apoptosis of the murine macrophages was detected by TUNEL staining. After washing with PBS, primary antibodies/markers were added: TUNEL staining solution (Roche, Germany) for cell apoptosis and anti-F4/80 antibody (1:200) (eBioscience) for macrophages in antibody dilution buffer at 37 °C for 1 h. After washing with PBS, cells were incubated for 30 min at RT with secondary antibodies prepared at 1:500 in antibody dilution buffer: Alexa 488 goat anti-rat IgG (Jackson ImmunoResearch Laboratories, Inc.). After secondary antibodies were removed and the tissues were washed again with PBS, nuclear counterstaining was performed by incubation with DAPI solution (Sigma-Aldrich Japan K.K., 1 µg/mL in PBS) for 10 min at RT. Images were examined using a fluorescent microscope (BZx-700, Keyence). TUNEL-positive cells in each chamber were counted in 5 different high power fields (×200). Apoptosis was evaluated by the TUNEL labeling index calculated as a TUNEL-positive percentage to total macrophage number.

### Quantitative Real-Time RT-PCR

Total RNA was extracted from lungs of mice after 21 days of receiving BLM with or without mAdSCs (n = 6 group). Murine activated macrophages were seeded onto 6-well Transwell culture plates and cultivated with or without mAdSCs for 4, 8, and 24 h at 37 °C in DMEM-F12 containing 1% FBS medium. mCD4+ T cells were seeded onto 6-well culture plates and cultivated in RPMI containing 10% FBS or mAdSC CM containing 10% FBS. Cells were harvested, and RNA was extracted with an RNeasy Mini Kit (Qiagen Ltd., Manchester, UK), cDNA was synthesized using an ExScript RT Kit (Takara, Shiga, Japan), and amplification was performed on a Sequence Detection System 7000 (Applied Biosystems) according to the manufacturer’s instructions. Primer sequences and GenBank accession numbers are as follows: tumor necrotic factor alpha (TNF-α, NM_013693): forward, TCTCCCTGATCGGTGACAGT and reverse, GGGCAGAGCTGAGTGTTAGC; interleukin 12 (IL-12, NM_008352): forward, AAGATGAAGGAGACAGAG and reverse, CATTGGACTTCGGTAGAT; GATA-binding protein-3 (GATA3, NM_008091): forward, TTATCAAGCCCAAGCGAAG and reverse, TGGTGGTGGTCTGACAGTTC; retinoic acid receptor-related orphan receptor-γt (Ror-γt, NM_001293734): forward, ACCTCTTTTCACGGGAGGA and reverse, TCCCACATCTCCCACATTG; forkhead box P3 (Foxp3, NM_001199347): forward, GAGAAAGTGGCAGAGAGGTA and reverse, CCACAGCATGGGTCTGTCT; collagen type 1 alpha 1 (COL1A1, NM_007742): forward, AAGAAGACATCCCTGAAG and reverse, ATACAGATCAAGCATACCT; and glyceraldehyde-3-phosphate dehydrogenase (GAPDH, 1137078): forward, ACAATGAATACGGCTACAG and reverse, GGTCCAGGGTTTCTTACT. Relative mRNA expression of the target gene was calculated with the comparative C_T_ method. The amount of the target gene was normalized to the endogenous GAPDH control gene. The experiments were run in triplicate, and the results were averaged.

### Statistical analysis

All values are presented as mean ± SEM. Comparisons between 2 groups were tested by Mann-Whitney U-test, and those among multiple groups were tested by significance via analysis of variance (ANOVA) followed by post-hoc testing with a Tukey procedure. The Kaplan-Meier method was used to assess survival curves, and the log-rank test was used to evaluate the significance of differences between the two groups. *P* values less than 0.05 were considered statistically significant. Statistical analyses were performed with commercially available software (GraphPad Prism, MDF Co. Ltd., Tokyo, Japan).

## Results

### mAdSCs Recruitment to Lung

**Figure 1:**
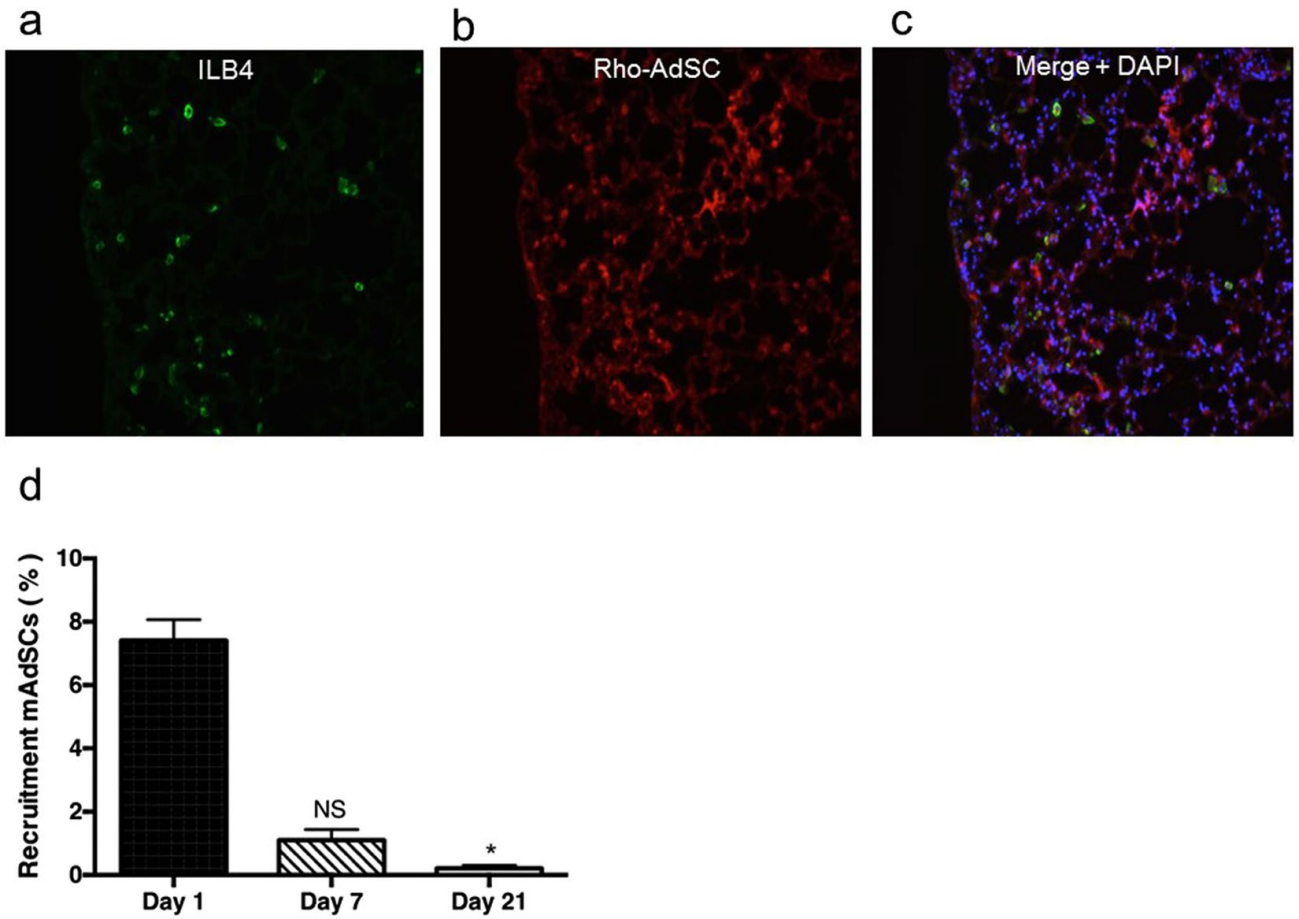
Recruitment of mAdSCs to lung. Recruitment of mAdSCs to lung was assessed using markers of ILB4 for endothelial cells and markers of rhodamine, which uptake into intracellular mitochondria, for mAdSCs. (**a**) Capillaries in the lung field stained by ILB4 (green). (**b**) mAdSCs stained by rhodamine (red) were observed in the interstitial space in the lung. (**c**) Nuclei were stained with DAPI (blue). A number of Rho-mAdSCs were observed in the interstitial spaces in the lung but not in the capillaries. (**d**) The percent of remained/detectable mAdSCs out of total cell number of homogenized injured lung tissue at different time points following mAdSC transplantation. Data are shown as mean ± SEM. *P < 0.05; NS, not significant vs. Day 1.

We assessed recruitment of mAdSCs to lung using markers of ILB4 for endothelial cells, and markers of rhodamine, which uptake into intracellular mitochondria, for mAdSCs. A number of Rho-mAdSCs were observed in the interstitial spaces in the lung (Fig. 1a–c). The recruited mAdSCs to the lung was quantified at different time points, and the percent of remained/detectable mAdSCs out of total cell number of homogenized injured lung tissue decreased time-dependently at 1, 7, and 21 days after mAdSC transplantation (Fig. 1d). These findings suggest that the recruited mAdSCs did not differentiate into other cell types in the injured lung, while they migrated to inflammatory sites of interstitium.

### mAdSCs Reduce Lung Inflammation

**Figure 2:**
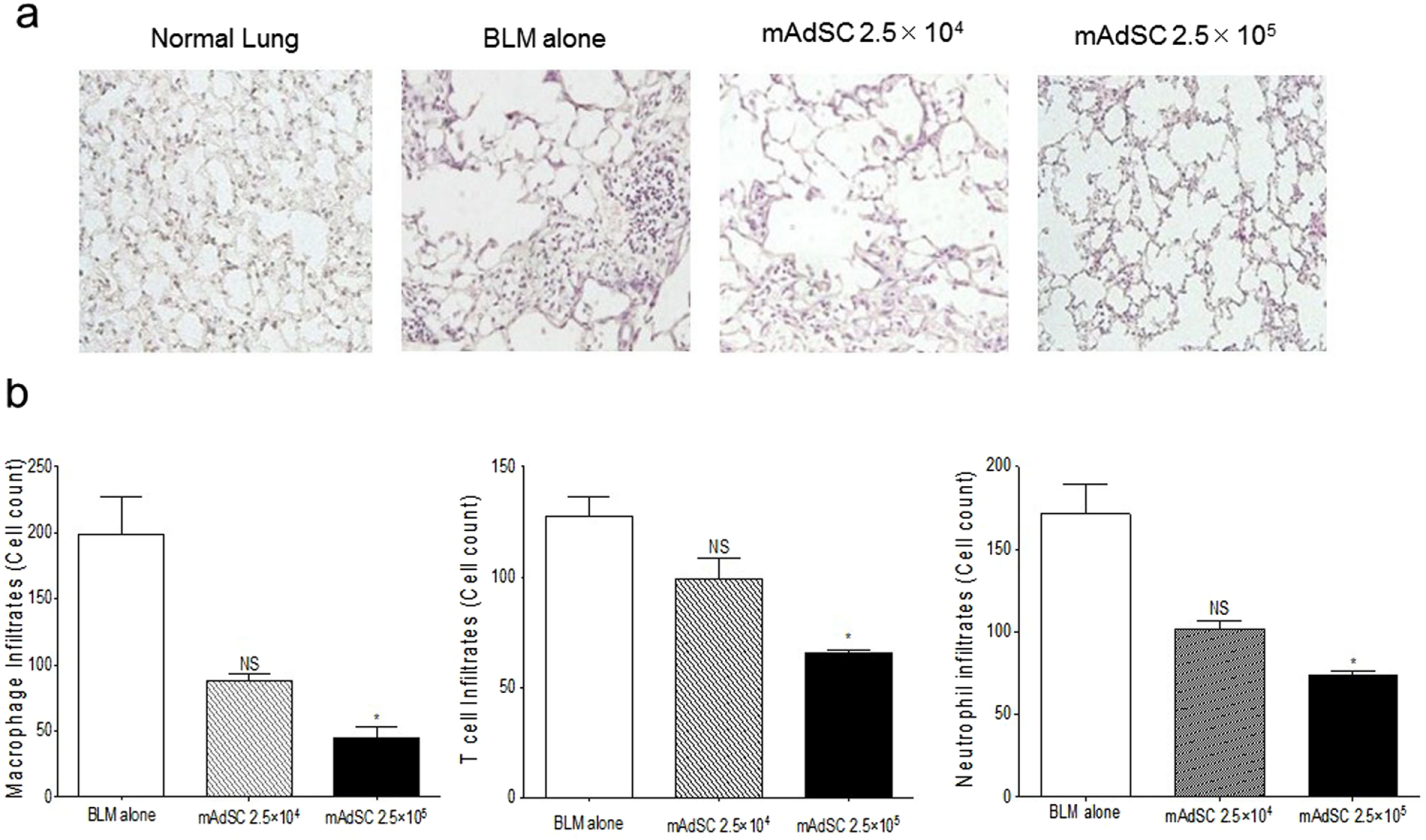
mAdSCs reduce lung inflammation. (**a**) Representative lung sections stained with H&E (×200) at 14 days after starting BLM administration (inflammation phase). A lung section from the BLM-alone group showed diffuse pneumonic lesions with loss of the normal alveolar architecture, septal thickening, enlarged alveoli, and increased infiltration of inflammatory cells in interstitial and peribronchiolar areas compared with normal lung. Both alveolar thickening and infiltration of inflammatory cells in the lung were reduced by administration of mAdSCs in a dose-dependent manner. (**b**) Analysis of infiltration of inflammatory cells in the lung fields showed that infiltration of macrophages, neutrophils, and T lymphocytes in the BLM-plus-mAdSCs (2.5 × 10^4^) and BLM-plus-mAdSCs (2.5 × 10^5^) groups were reduced in comparison with that in the BLM-alone group and was especially significantly reduced in the BLM-plus-mAdSCs (2.5 × 10^5^) group. Data are shown as mean ± SEM. *P < 0.05; **P < 0.01, NS, not significant vs. BLM-alone group.

Histological evaluations using H&E staining of the lung at 14 days after starting BLM injection (inflammation phase) are shown in Fig. 2a (the raw data were presented in Supplementary Fig. S3). Diffuse pneumonic lesions with loss of the normal alveolar architecture, septal thickening, enlarged alveoli, and increased infiltration of inflammatory cells in interstitial and peribronchiolar areas were revealed in BLM-IP lung compared with normal lung. Both alveolar thickening and infiltration of inflammatory cells in the lung were reduced in BLM-plus-mAdSCs lung in a dose-dependent manner. Analysis of infiltration of inflammatory cells in the lung fields showed that infiltrations of macrophages, neutrophils, and T lymphocytes in the BLM-plus-mAdSCs (2.5 × 10^4^) and BLM-plus-mAdSCs (2.5 × 10^5^) groups were reduced in comparison with those in the BLM-alone group and were especially significantly reduced in the BLM-plus-mAdSCs (2.5 × 10^5^) group (Fig. 2b–d).

### mAdSCs Reduce Lung Fibrosis

**Figure 3:**
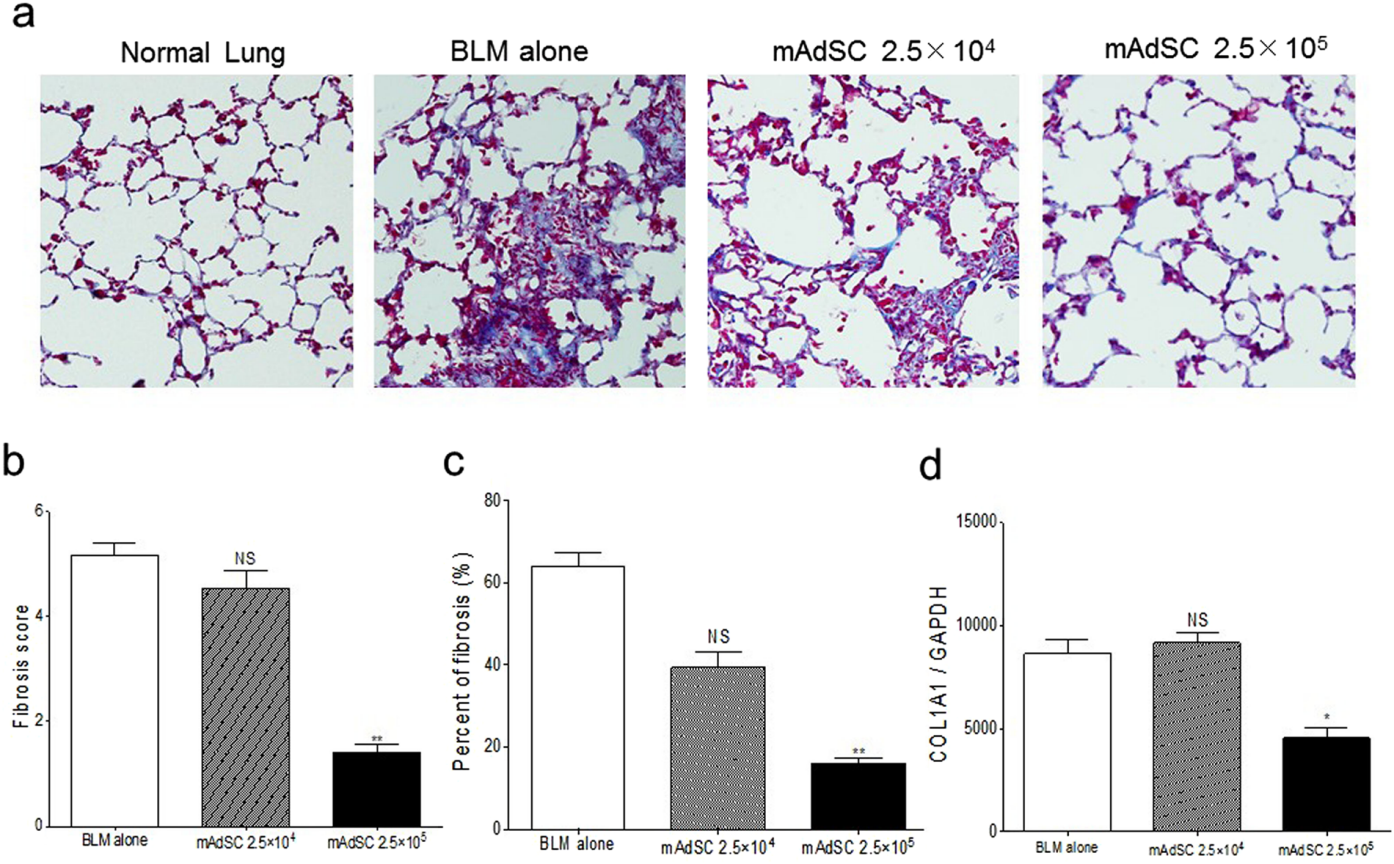
mAdSCs reduce lung fibrosis. (**a**) Representative lung sections stained with Masson’s trichrome staining (×200) at 28 days after starting BLM administration (fibrosis phase). A lung section from the BLM-alone group showed the diffuse increase of collagen deposition compared with normal lung. The increase in collagen deposition in the lung was reduced by administration of mAdSCs in a dose-dependent manner. (**b**) The fibrosis score was significantly lower in the BLM-plus-mAdSCs (2.5 × 10^5^) group (P < 0.01) but was not lower in the BLM-plus-mAdSCs (2.5 × 10^4^) group than that in the BLM-only group. (**c**) The ratio of fibrotic area to total area was significantly lower in the BLM-plus-mAdSCs (2.5 × 10^5^) group (P < 0.01) but was not lower in the BLM-plus-mAdSCs (2.5 × 10^4^) group than that in the BLM-only group. (**d**) There was an increase in COL1A1 mRNA expression at 28 days following BLM administration. Although the levels of COL1A1 mRNA expression at 28 days after starting BLM administration were not lower in BLM-plus-mAdSCs (2.5 × 10^4^) group, they were significantly lower in the BLM-plus-mAdSCs (2.5 × 10^5^) group than those in the BLM-alone group (P < 0.05). Data are shown as mean ± SEM. *P < 0.05; **P < 0.01, NS, not significant vs. BLM-alone group.

Increased collagen deposition caused fibrosis in BLM-IP lung, and mAdSC administration reduced the fibrosis in a dose-dependent manner (Fig. 3a). The fibrosis score and the ratio of fibrotic area to total area were significantly lower in the BLM-plus-mAdSCs (2.5 × 10^5^) group but not lower in the BLM-plus-mAdSCs (2.5 × 10^4^) group than those in the BLM-only group (Fig. 3b,c). To determine if the fall in collagen was due to reduced synthesis, whole lung mRNA expression was analyzed by quantitative real-time PCR for COL1A1. There was an increase in COL1A1 mRNA expression at 28 days following BLM administration. Although the levels of COL1A1 mRNA expression at 28 days after starting BLM administration were not lower in the BLM-plus-mAdSCs (2.5 × 10^4^) group, they were significantly lower in the BLM-plus-mAdSCs (2.5 × 10^5^) group than those in the BLM-alone group (Fig. 3d).

### Intravenous Injection of mAdSCs Improves Prognosis in BLM-IP Mice

**Figure 4:**
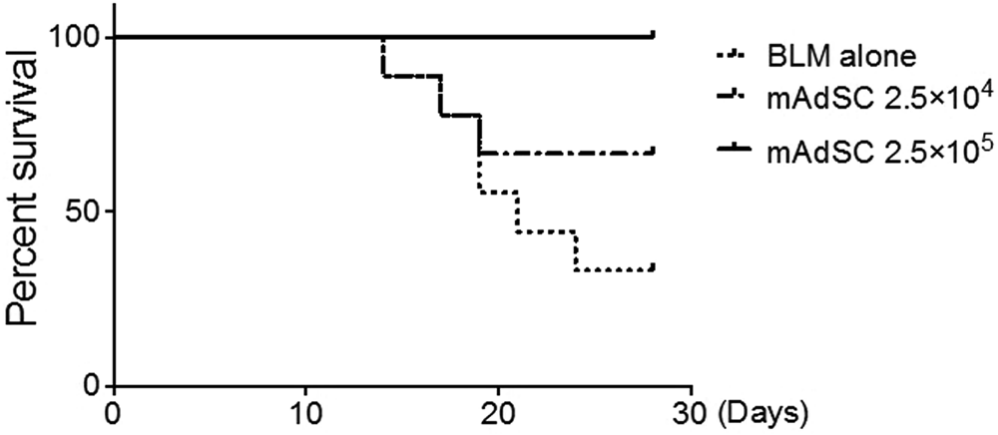
Intravenous injection of mAdSCs improves the prognosis in BLM-IP mice. Evaluation of survival curves comparing the BLM-alone group and the BLM-plus-mAdSCs group showed that 67% of the mice in the BLM-alone group and 33% in the BLM-plus-mAdSCs (2.5 × 10^4^) group died within 28 days. Contrastingly, all mice in the BLM-plus-mAdSCs (2.5 × 10^5^) group survived up to 28 days.

Survival curves were evaluated between the BLM-alone group and the BLM-plus-mAdSCs group (Fig. 4). Sixty-seven percent of the mice in the BLM-alone group and 33% in the BLM-plus-mAdSCs (2.5 × 10^4^) group died within 28 days. Strikingly, all mice in the BLM-plus-mAdSCs (2.5 × 10^5^) group survived up to 28 days.

### Effects of mAdSC on Activated Murine Macrophages

**Figure 5:**
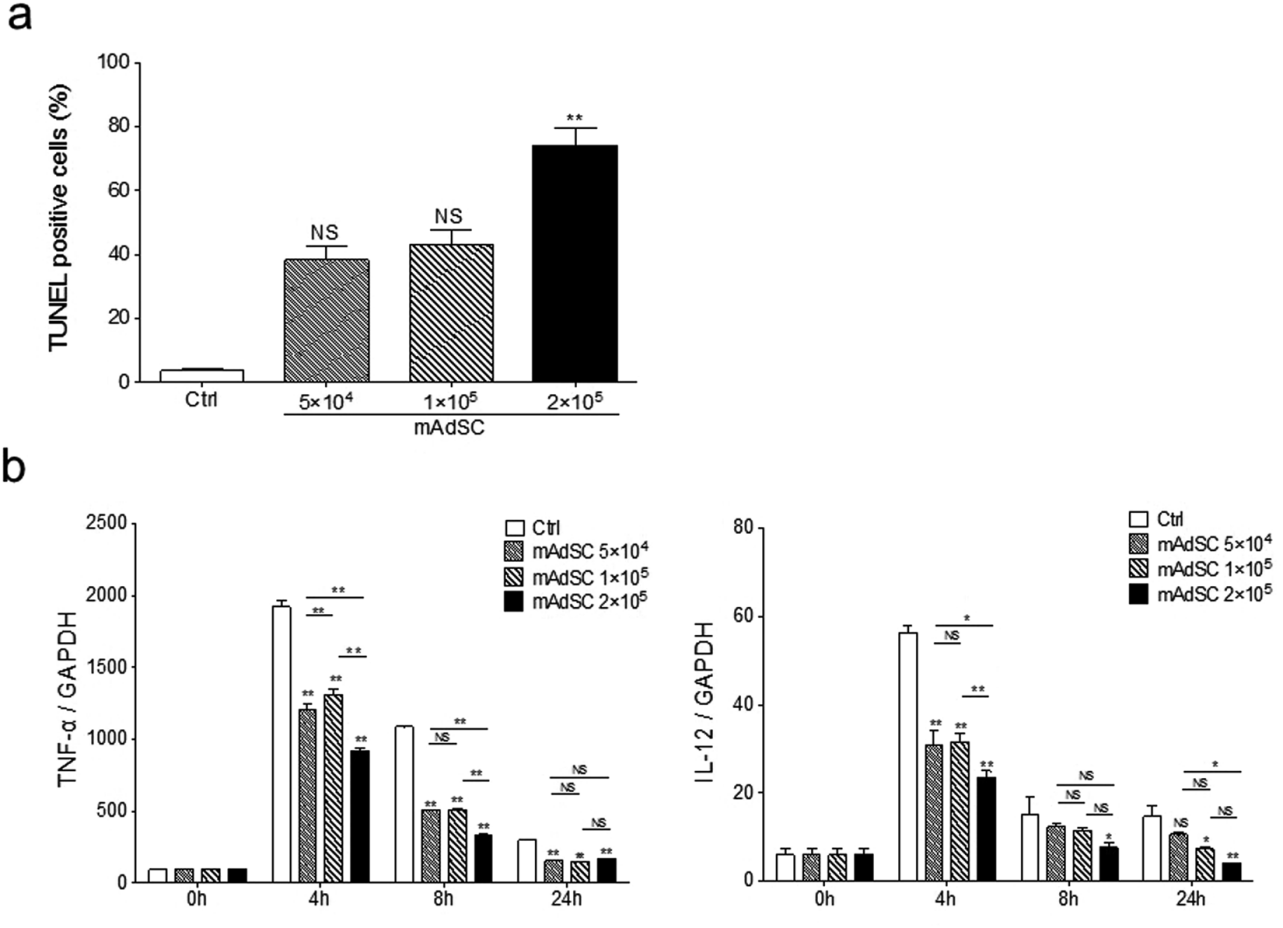
Effects of mAdSC on activated murine macrophages *in vitro*. (**a**) The number of TUNEL-stained positive macrophages was significantly increased in the mAdSCs (2.0 × 10^5^) group compared with the control group (P < 0.01). There was no significant difference among the mAdSCs (1.0 × 10^5^) group, the mAdSCs (5.0 × 10^4^) group, and control group. (**b**) The production of cytokines in activated macrophages was evaluated by quantitative real-time RT-PCR. TNF-α and IL-12 mRNA expressions in macrophages co-cultured with mAdSCs were significantly down-regulated in a dose-dependent manner. *P < 0.05; **P < 0.01; NS, not significant vs. control group. All experiments were performed in triplicate and statistically analyzed.

Macrophage apoptosis was assessed by TUNEL staining. The number of apoptotic macrophages was significantly increased in the mAdSCs (2.0 × 10^5^) group compared with the control group, but there was no significant difference among the mAdSCs (1.0 × 10^5^) group, the mAdSCs (5.0 × 10^4^) group, and the control group (Fig. 5a). The production of cytokines in activated macrophages was evaluated by quantitative real-time RT-PCR. TNF-α and IL-12 mRNA expressions in macrophages co-cultured with mAdSCs were significantly down-regulated in a dose-dependent manner (Fig. 5b). These results suggest that mAdSCs induced apoptosis of activated macrophages, and the anti-inflammatory cytokine synthesis was inhibited in activated macrophages by co-culture with mAdSCs.

### Effects of mAdSC on Murine CD4+ T Cells

**Figure 6:**
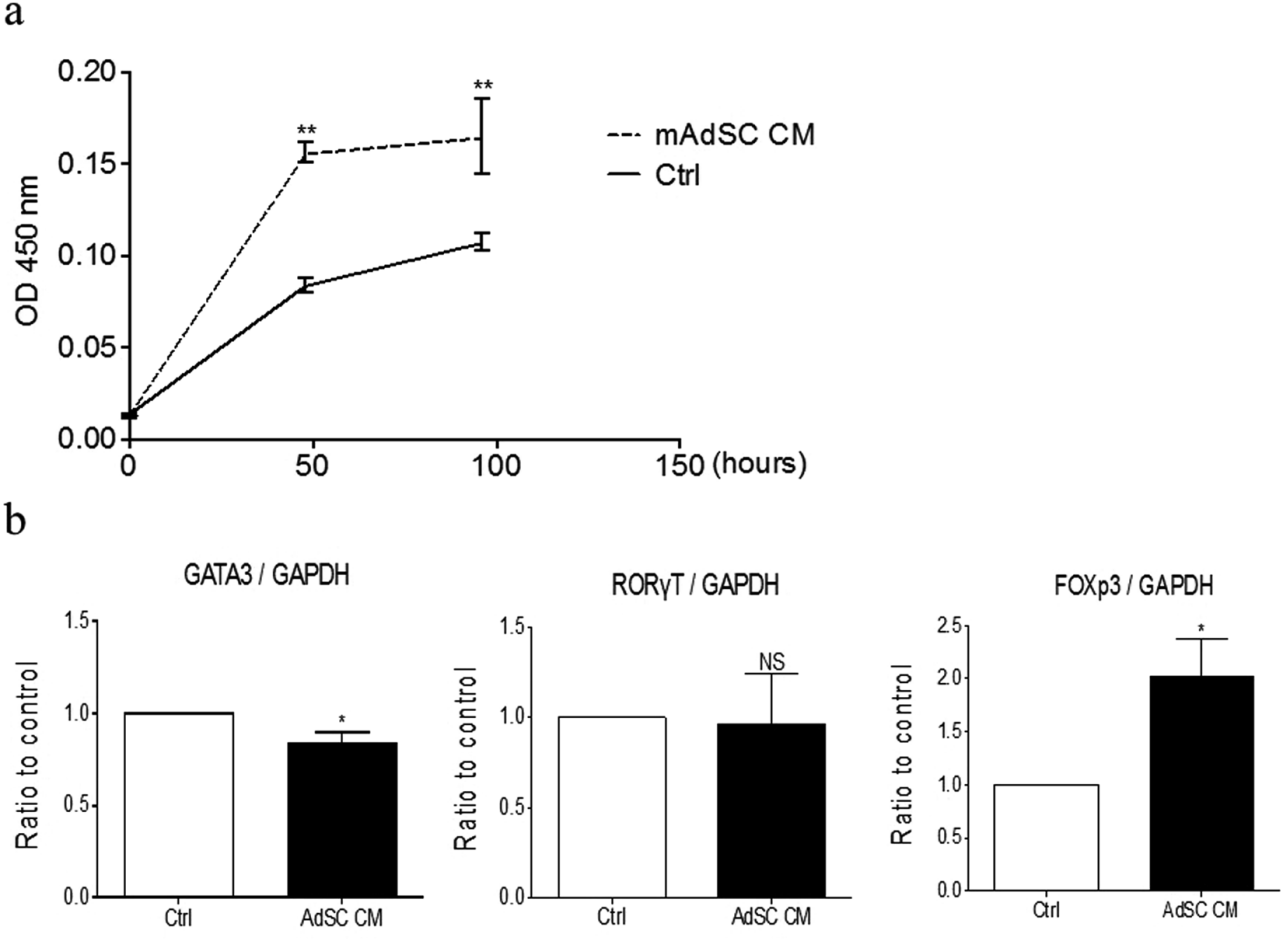
Effects of mAdSC on murine CD4+ T cells *in vitro*. (**a**) The proliferation activity of mCD4+ T cells is expressed as the mean optical density value at a wavelength of 450 nm. mCD4+ T cells were incubated for 96 h with 10% FBS-RPMI medium or mAdSC CM. mAdSC CM had a significant effect on the proliferation activity of mCD4+ T cells. (**b**) The gene expression of GATA-binding protein-3 (GATA3), retinoic acid receptor-related orphan receptor-γt (Ror-γt), and forkhead box P3 (Foxp3) was determined in mCD4+ T cells that were incubated for 48 h with 10% FBS-RPMI medium (control) or mAdSC CM. mAdSC CM significantly increased the Foxp3 and decreased the GATA3 gene expression ratio to control (both, P < 0.05), but the ratio for Ror-γt was not changed. GATA3, Ror-γt, and Foxp3 have been defined as the master regulators of Th2 cells, Th17 cells, and regulatory T cells, respectively. CM, condition medium; *P < 0.05; **P < 0.01; NS, not significant vs. control group. All experiments were performed in triplicate and statistically analyzed.

The proliferation activity of mCD4+ T cells is expressed as the mean optical density value at a wavelength of 450 nm. mCD4+ T cells were incubated for 96 h with 10% FBS-RPMI medium or mAdSC CM. mAdSC CM had a significant effect on the proliferation activity of the mCD4+ T cells (Fig. 6a).

The gene expression of GATA3, Ror-γt, and Foxp3 was examined in mCD4+ T cells that were incubated for 48 h with 10% FBS-RPMI medium (control) or mAdSC CM. mAdSC CM significantly increased the Foxp3 and decreased the GATA3 gene expression ratio compared to the control (P = 0.029 and 0.043, respectively), but the ratio was unchanged for ROR-γT (Fig. 6b). These results suggest that mAdSCs inhibit the differentiation and proliferation of Th2-type T cells of mCD4+ T cells and promote the differentiation and proliferation of regulatory T cells (T-reg).

## Discussion

In the present study, intravenous mAdSCs markedly improved the survival rate of mice with BLM-induced IP that developed diffuse lesions in the lung in a dose-dependent manner by accumulating in the lung interstitium and inhibiting both pulmonary inflammation and fibrosis. mAdSCs inhibited the production of pro-inflammatory cytokines, such as TNF-α and IL-12 in activated macrophages, and mAdSCs also induced the apoptosis of activated macrophages. In addition, mAdSCs inhibited the differentiation and proliferation of Th2-type T cells of mCD4+ T cells, suggesting that mAdSCs promote the differentiation and proliferation of T-reg and is thus the mechanism of inhibition of pulmonary inflammation by mAdSCs.

Three studies on the efficacy of mAdSCs in mice with BLM-induced IP have been reported. Lee *et al*. prepared IP mice by the airway administration of BLM to 8-week-old B6 mice, intraperitoneally administered 3 × 10^5^ human mAdSCs to these mice 4 times every 2 weeks from 8 weeks after BLM administration, and observed that pulmonary fibrosis and lung tissue apoptosis were inhibited^27^. Tashiro *et al*. prepared IP mice by the airway administration of BLM to 22-week-old B6 mice, intravenously injected 5 × 10^5^ mAdSCs collected from 4-week-old (young) or 22-week-old (elderly mice) once one day after BLM administration, and investigated the inhibition of pulmonary fibrosis. The inhibitory effect on pulmonary fibrosis of mAdSCs from the young mice was superior to that from the old mice^28^. Rathinasabapathy *et al*. prepared IP mice by the airway administration of BLM to 8-week-old mice, intravenously administered 1 × 10^6^ mAdSCs collected from 8–9-week-old mice and mAdSC-conditioned medium once at 3 (early phase) or 7 (late phase) days after BLM administration, and observed that pulmonary fibrosis was inhibited^29^. These reports suggest that mAdSCs and mAdSC-conditioned medium effectively inhibit pulmonary fibrosis in BLM-induced IP mice, thus supporting our findings. The number of cells capable of exhibiting the effect of mAdSCs, the effect on the inflammatory phase, or the accumulation of mAdSCs in lung tissue in BLM-induced IP mice was not investigated in these previous studies. We confirmed that mAdSCs exhibited the effect in a dose-dependent manner, and the number of cells required to exhibit the effect was 2.5 × 10^5^, which was the lowest compared with previously reported numbers. In addition, it was confirmed that mAdSCs also inhibited pulmonary inflammation in the pulmonary inflammatory phase in the BLM-induced IP mice.

We administered mAdSCs 3 days after initiation of BLM treatment and investigated the effect on pneumonia-inhibiting (Supplementary Fig. S4). mAdSCs inhibited pulmonary inflammation in a dose-dependent manner, but the inhibition was stronger in the group treated with mAdSCs after completion of BLM administration. The reason for this is unclear, but it is possible that BLM toxicity impaired AdSC function when they were administered during the BLM administration period in this model. It was also considered that although AdSCs were administered in the inflammation phase of IP, the recruited AdSCs to highly inflammatory lung tissue might be negatively affected by inflammatory cytokines. Although higher therapeutic efficacy may be obtained by administering AdSCs in an early stage of disease in clinical settings, further investigation is necessary for its clinical application.

In a study with a mouse model of pulmonary emphysema, human AdSCs were intravenously injected into the tail vein, and accumulation of AdSCs in the lung was evaluated after 1, 7, and 21 days. AdSCs accumulated most markedly in the lung interstitium one day after intravenous injection^33^. Similarly, mAdSC accumulation in the lung interstitium was noted one day after intravenous injection in our study, initially confirming mAdSC accumulation in the lung interstitium in the BLM-IP mice.

MSCs have an inhibitory action by inhibiting inflammatory cytokine production by macrophages^34^. AdSCs inhibited gene expression in M1 macrophages and induced gene expression in M2 macrophages in macrophage cell lines. In an *in vivo* study, mAdSCs inhibited activated macrophages through an immunomodulatory ability in a mouse model of inflammatory bowel disease^35^. mAdSCs also had an inhibitory effect on nephritis by converting macrophages to immunoregulatory cells in a glomerulonephritis mouse model^27^. Actually, we confirmed that mAdSCs had an inhibitory effect on activated macrophages.

Yousefi *et al*. co-cultured mAdSCs collected from B6 mice and their conditioned medium with naive T cells *in vitro* and observed that T-reg were induced^36^. They also intravenously injected mAdSCs into mice in an experimental autoimmune encephalomyelitis model and observed that the T-reg population increased in the spleen. Furthermore, Xie *et al*. reported that mAdSCs induced M2 macrophages and the T-reg phenotype in a manner dependent on the number of cells *in vitro* and also increased T-reg and M2 macrophages and decreased the accumulation of CD4 + CD28− and CD8 + CD28− T cells and Ly6G/C+ neutrophils in lesions in a mouse model of abdominal aortic aneurysm prepared with B6 mice^37^. In the respiratory field, Cho *et al*. reported that mAdSCs inhibited Th2 cytokines (IL-4, IL-5, and IL-13) and increased the expression of immunosuppressive system cytokines (IL-10 and TGF-b1) in the intrapulmonary lymph nodes of asthmatic mice^38^, and they also reported that mAdSCs significantly increased T-reg and IL-10+ T-cell populations and induce T-reg differentiation through PGE2 and TGF-b1^39^. These findings suggest that mAdSCs and their conditioned medium have an inhibitory effect on inflammatory diseases directly or through the mechanism inducing T-reg through PGE2 and TGF-b1 and inhibiting Th2 cytokines. Similarly, mAdSC-conditioned medium induced T-reg and inhibited Th2-type T cells in our study, suggesting that this is a mechanism leading to the effectiveness of mAdSCs in the BLM-induced IP mouse model, which is a pathological condition of the Th2 immune response.

A high mortality rate of up to 85% was documented in the mice following the intravenous infusion of mAdSCs within 24 h due to the observation of pulmonary embolism. As the mechanism of pulmonary embolism, it has been suggested that tissue factor is expressed on the mAdSC surface and triggers procoagulation^40^. No death of a mouse considered to be due to pulmonary embolism was noted in our study, and this may have been due to the small number of cells injected intravenously at a slow rate while inhibiting cell metabolism by cooling mAdSCs 4 °C before administration.

There are several limitations in this study. The mouse BLM-IP model phenotype is similar to human acute lung injury/acute respiratory distress syndrome (ARDS) in acute inflammatory phase, but fibroblastic foci, alveolar epithelial type 2 cells hyperplasia, and honeycombing lesions were reduced compared with those in humans, indicating that the reproduction of human IP was not complete in mouse BLM-IP. In this study, mAdSCs were administered at 7 days after initiation of BLM when fibrosis was not developed, and several studies reported that MSC administration did not improve the pathologically established pulmonary fibrosis^14,41,42^. Regarding the efficacy of mAdSCs in the fibrosis phase, further studies are necessary and our investigation is ongoing. As the reviewer stated, some studies suggest that MSCs exacerbate pulmonary fibrosis^11,12,41^. It is unclear whether MSCs promote pulmonary fibrosis, but the lack of effect may be due to differences of fibrosis stage after induction, species of model animals, and administration of MSCs in the fibrosis growth phase rather than in the inflammatory phase. Because the IP-specific cellular and molecular mechanisms with MSCs have not been sufficiently clarified yet, clinical application should be considered carefully.

## Conclusions

Intravenous mAdSCs accumulated in the lung interstitium and inhibited both pulmonary inflammation and fibrosis, which markedly improved the survival rate of mice with BLM-induced lung injury in a dose-dependent manner. The functional inhibition of activated macrophages and T-reg induction by mAdSCs were considered to be its mechanisms.

## Electronic supplementary material

Supplemental file
