# Publisher Correction: Anti-inflammatory and anti-fibrotic effects of intravenous adipose-derived stem cell transplantation in a mouse model of bleomycin-induced interstitial pneumonia

**DOI:** 10.1038/s41598-017-17991-x

**Published:** 2018-01-08

**Authors:** Takuya Kotani, Ryota Masutani, Takayasu Suzuka, Katsuhiro Oda, Shigeki Makino, Masaaki Ii

**Affiliations:** 1 Department of Internal Medicine (IV), Osaka Medical College, Osaka, Japan; 2 Division of Research Animal Laboratory and Translational Medicine, Research and Development Center, Osaka Medical College, Osaka, Japan; 3 Division of Central Laboratory, Osaka Medical College, Osaka, Japan

Correction to: *Scientific Reports* https://doi.org/10.1038/s41598-017-15022-3, published online 03 November 2017

The original HTML version of this Article contained a typographical error in the publication date ‘03 November 2017’ which was incorrectly given as ‘06 November 2017’. This has now been corrected in the HTML version of the Article.

