# Publisher Correction: Anti-inflammatory and anti-fibrotic effects of intravenous adipose-derived stem cell transplantation in a mouse model of bleomycin-induced interstitial pneumonia

**DOI:** 10.1038/s41598-018-22374-x

**Published:** 2018-03-06

**Authors:** Takuya Kotani, Ryota Masutani, Takayasu Suzuka, Katsuhiro Oda, Shigeki Makino, Masaaki Ii

**Affiliations:** 1 Department of Internal Medicine (IV), Osaka Medical College, Osaka, Japan; 2 Division of Research Animal Laboratory and Translational Medicine, Research and Development Center, Osaka Medical College, Osaka, Japan; 3 Division of Central Laboratory, Osaka Medical College, Osaka, Japan

Correction to: *Scientific Reports* 10.1038/s41598-017-15022-3, published online 03 November 2017

An error occurred after publication resulting in the inadvertent omission of the Article and Volume numbers from the PDF version of this Article. This error has now been corrected in the PDF version of the Article; the HTML version was correct from the time of publication.

