# Supplementary material for: Anti-inflammatory and anti-fibrotic effects of intravenous adipose-derived stem cell transplantation in a mouse model of bleomycin-induced interstitial pneumonia
Source: Sci Rep. 2017 Nov 3;7:14608. doi: 10.1038/s41598-017-15022-3 (PMC5668313; doi:10.1038/s41598-017-15022-3)
Supplement: Supplementary file 1 — Supplemental file [file 41598_2017_15022_MOESM1_ESM.pdf]

Supplementary Information for:

Anti-inflammatory and anti-fibrotic effects of intravenous adipose-derived stem cell transplantation in a mouse model of bleomycin-induced interstitial pneumonia

Takuya Kotani[1], Ryota Masutani[3], Takayasu Suzuka[1], Katsuhiro Oda[1], Shigeki Makino[1], Masaaki Ii[2*]

[1]Department of Internal Medicine (IV), Osaka Medical College, Osaka, Japan

[2]Division of Research Animal Laboratory and Translational Medicine, Research and Development Center, Osaka Medical College, Osaka, Japan

[3]Division of Central Laboratory, Osaka Medical College, Osaka, Japan

*Corresponding author: Masaaki Ii, MD, PhD

Division of Research Animal Laboratory and Translational Medicine, Research and Development Center, Osaka Medical College

2-7 Daigaku-machi, Takatsuki, Osaka 569-8686, Japan

Tel: +81-72-684-6537, Fax: +81-72-684-7042

E-mail: masaii@osaka-med.ac.jp

This supplement contains:

Supplementary Figure S1

Supplementary Fig. S1. Fluorescent signals of Rho-mAdSCs in culture.

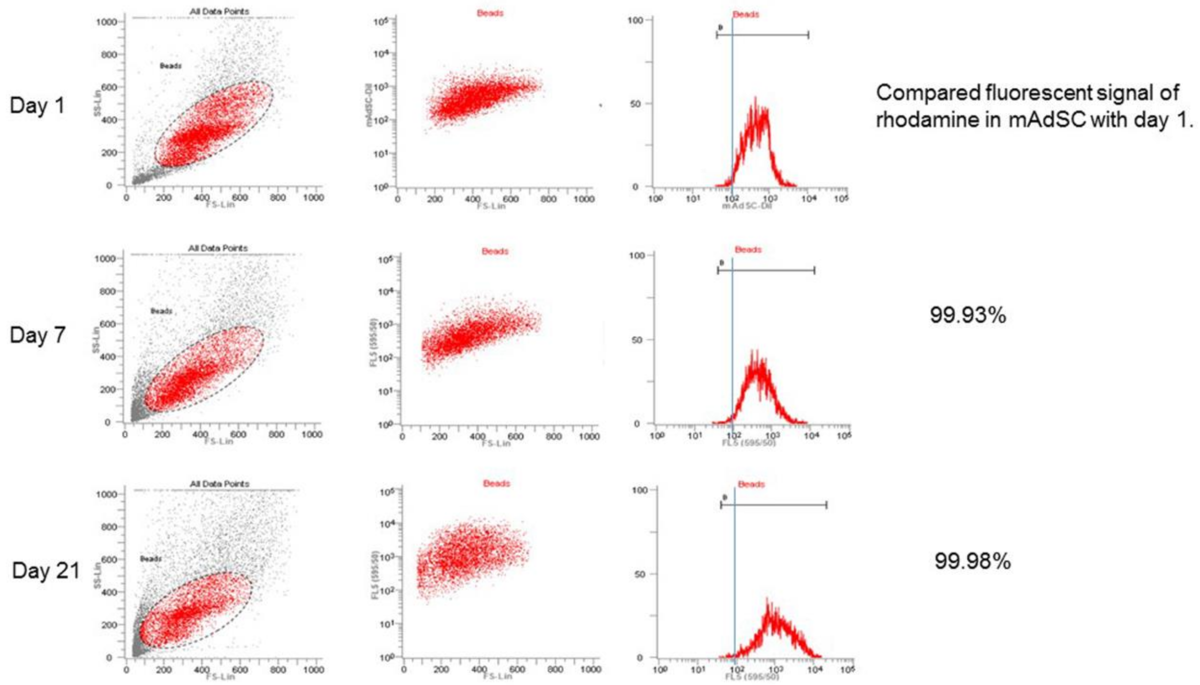

The fluorescent signals of Rho-mAdSCs were analyzed on day 1, 7, and 21 after Rhodamine-PLGA nanoparticle uptake and culture by flow cytometry. The percent of red fluorescent-positive cells on day 7 (99.93%) and day 21 (99.98%) were similar to that on day 1. AdSC: adipose-derived stem cell, Rho-mAdSCs: rhodamine-PLGA-nanoparticle loaded mAdSCs.

This supplement contains:

Supplementary Figure S2

Supplementary Fig. S2. AdSC transfusion study protocol.

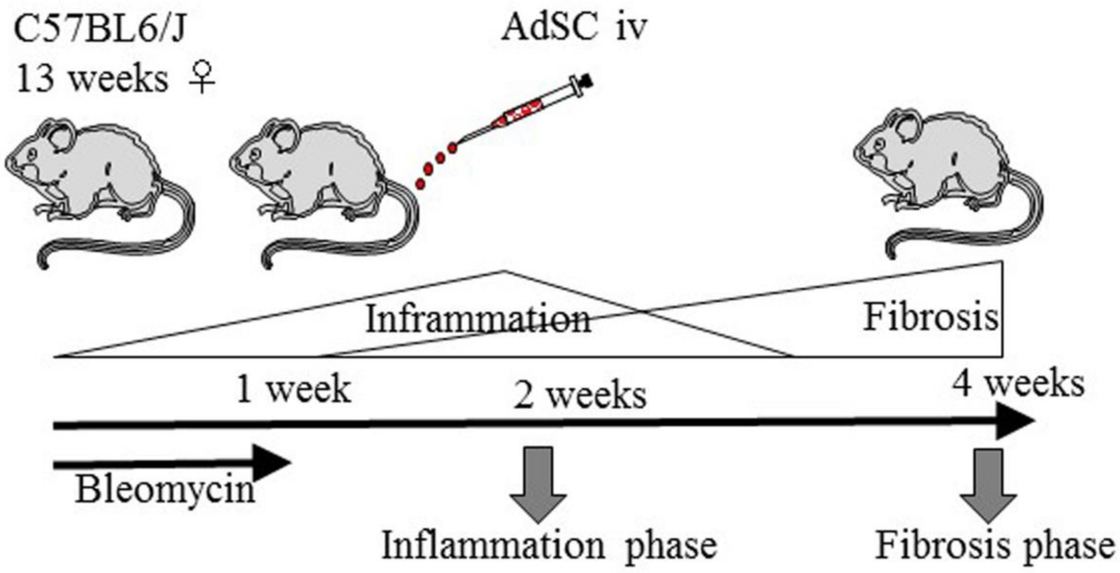

AdSC iv: intravenous adipose-derived stem cell.

This supplement contains:

Supplementary Figure S3

Supplementary Fig. S3. The raw data of histological evaluations using H&E staining of the lung at 14 days after starting BLM injection (magnification ×100).

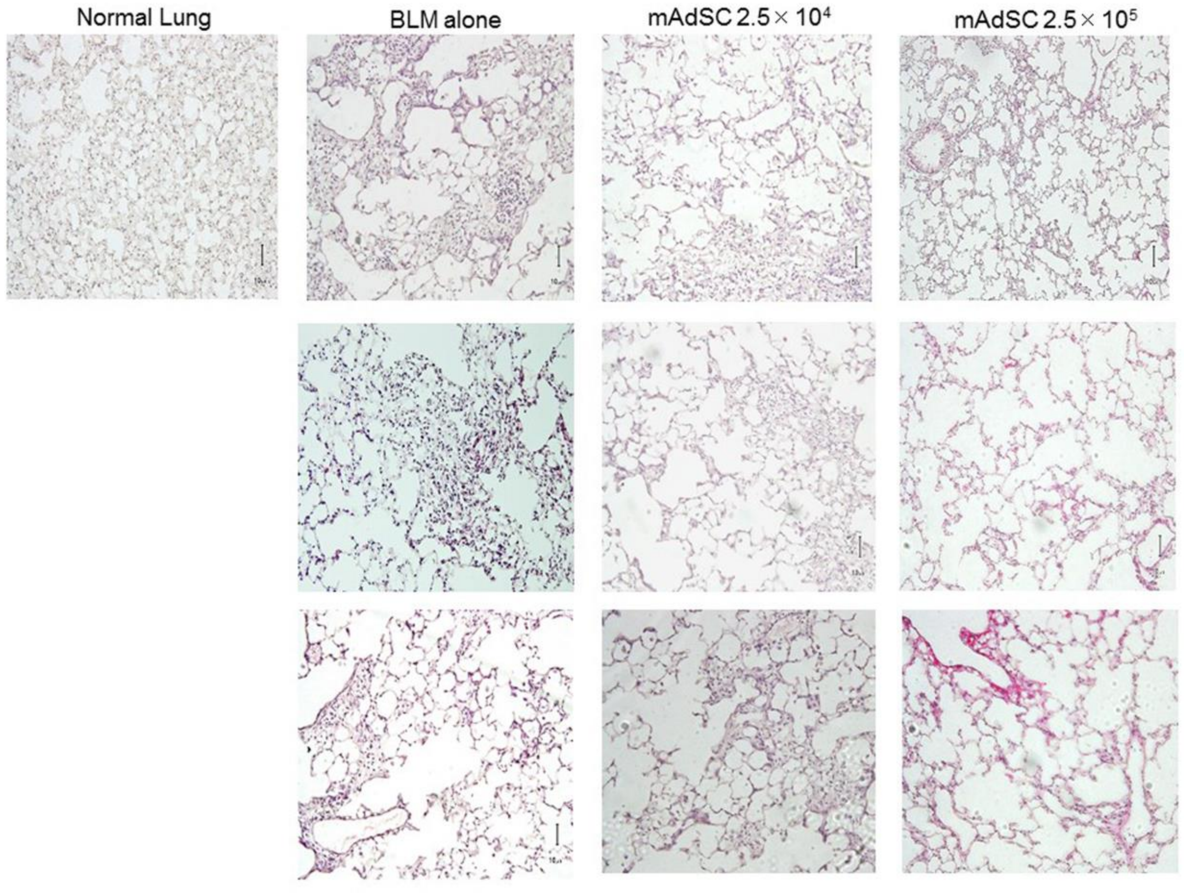

This supplement contains:

Supplementary Figure S4

Supplementary Fig. S4. Histological analysis of BLM-treated lung with or without mAdSC transplantation in early phase of inflammation.

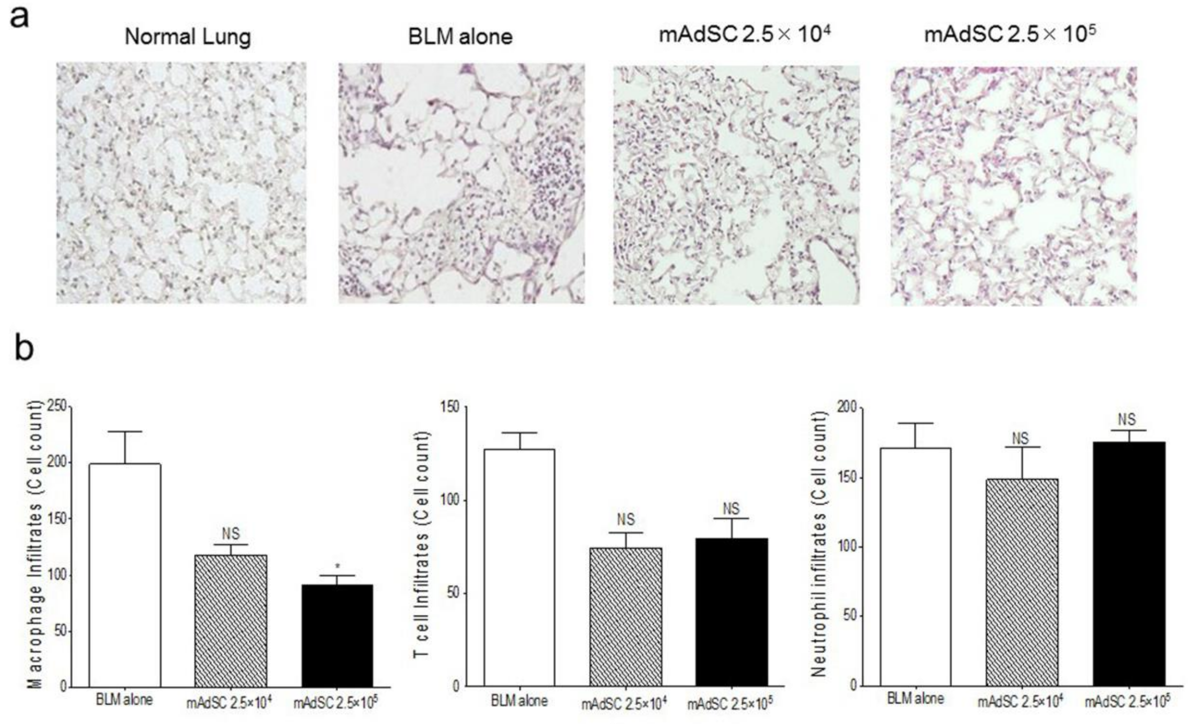

Representative lung tissue sections stained with H&E (×200) (a) and quantitative analysis of infiltration of inflammatory cells in the lung fields (b) at 14 days after starting BLM administration (inflammation phase). After starting administration of BLM, mAdSCs were infused via a tail vein on day 3. mAdSC 2.5×10^4, BLM with mAdSCs (2.5×10^4) group. mAdSC 2.5×10^5, BLM with mAdSCs (2.5×10^5) group. Data are shown as mean ± SEM. *P

< 0.05. NS, not significant vs. BLM-alone group.
